# Spatially Interpolated Disease Prevalence Estimation Using Collateral Indicators of Morbidity and Ecological Risk

**DOI:** 10.3390/ijerph10105011

**Published:** 2013-10-14

**Authors:** Peter Congdon

**Affiliations:** School of Geography, Queen Mary University of London, London E1 4NS, UK; E-Mail: p.congdon@qmul.ac.uk; Tel.: +44-20-7882-8200

**Keywords:** disease prevalence, spatial interpolation, neighbourhoods, asthma, kernel

## Abstract

This paper considers estimation of disease prevalence for small areas (neighbourhoods) when the available observations on prevalence are for an alternative partition of a region, such as service areas. Interpolation to neighbourhoods uses a kernel method extended to take account of two types of collateral information. The first is morbidity and service use data, such as hospital admissions, observed for neighbourhoods. Variations in morbidity and service use are expected to reflect prevalence. The second type of collateral information is ecological risk factors (e.g., pollution indices) that are expected to explain variability in prevalence in service areas, but are typically observed only for neighbourhoods. An application involves estimating neighbourhood asthma prevalence in a London health region involving 562 neighbourhoods and 189 service (primary care) areas.

## 1. Introduction

Geographic data on disease prevalence are important for assessing health inequities within regions, but are not necessarily collected for small areas or neighbourhoods suitable for health profiling. However, data on disease prevalence may be collected by health service providers for the populations they serve. Thus in the UK, prevalence data on chronic diseases is collected by general practitioner (GP) teams responsible for primary care. The population cared for by each GP team (subsequently called GP areas) is typically dispersed over several neighbourhoods. This raises the question whether data on prevalence for GP areas (source areas) can be used to provide spatially interpolated estimates for neighbourhoods (target areas) [1]. 

Many interpolation strategies rely simply on geographic location data for interpolating values to target areas, but additional information relevant to interpolation may be available. There may be observed data for neighbourhoods on morbidity outcomes (e.g., hospital admissions) providing ancillary information on neighbourhood prevalence. Ecological risk factor data (e.g., air quality) relevant to explaining prevalence variations may also be available. In the terminology of structural equation modeling there are both reflexive indicators (morbidity indicators), and formative indicators (ecological covariates), relevant to the interpolation process [2]. The analysis here extends that of Congdon [3] which considered situations involving only reflexive indicators. The analysis here is also distinctive in considering situations where the formative indicators are only observed for one spatial framework, and must be interpolated to the other.

The approach used here to take account of such collateral information is based on a technique known as discrete process convolution [4]. This method involves convolution of a random effect process over a discrete grid with a smoothing kernel to estimate the underlying spatial trend. The random effects are sampled at points in a regular grid covering the region. Whereas geostatistical interpolation using multivariate normal approaches tend to be computationally demanding as the number of source or target regions increases [5], the convolution method can be applied with large numbers of regions (e.g., over 500) in either or both source and target frameworks. 

Typically two forms of interpolation are required. Prevalence estimation for neighbourhoods (the target areas) necessitates spatial interpolation, since prevalence is actually only observed for service areas (GP areas). Additionally, ecological covariates are typically only available for neighbourhoods. In order to assess links between prevalence and ecological covariates over GP areas, spatial interpolation of ecological covariates (formative indicators) to these areas is required.

The analysis involves comparison of different forms of kernel and discrete grid random effects. A Bayesian estimation method is applied via repeated sampling (Markov chain Monte Carlo or MCMC) methods. Both fit of the model to the observed data, and the characteristics of the spatially interpolated disease estimates, may be sensitive to kernel choice or the random effect process assumed. In applications (as here) involving formative indicators, there may be sensitivity to additional modeling choices, for example, linear or nonlinear effects of ecological covariates on disease prevalence. 

## 2. Case Study Application

The case study setting is provided by a particular region within England (the largest country within the UK), where population prevalence registers for a range of chronic diseases are maintained by 8,200 GP teams under a system called the Quality Outcomes Framework (QOF). In the UK, primary care is provided by general practitioners from community based clinics, and either involves groups of GPs (and ancillary staff such as nurses) based in the same clinic, or single practitioners. The chronic prevalence data are provided only for such GP teams, and not for any small area populations. The application is to a health region within London, namely four London boroughs (Barking & Dagenham, Havering, Redbridge, Waltham Forest) collectively denoted as Outer North East London, and with a population of 970,000 in 2012. 

There are 189 GP practice areas (source areas), and 562 neighbourhoods (target areas), in this part of London. Neighbourhoods are defined as Lower Level Super Output Areas (LSOAs), of which there are currently 32,844 across England, with an average of 1,500 residents and 650 households. These are derived from smaller Census output areas [6], subject to constraints of proximity (to ensure a compact shape), and social homogeneity within each LSOA (e.g., similar household tenure and dwelling type patterns). 

It is important to note that data for GP service areas cannot be obtained by aggregating over neighbourhoods. There is no simple nested area hierarchy of neighbourhoods within GP service areas. In the UK in general, and the study region in particular, a GP service area is typically diffuse and scattered over several neighbourhoods. Conversely people in any particular neighbourhood can choose between GP teams and are typically split between several GP teams. 

The analysis here uses data in one or other framework and interpolates to the other. For example, interpolated prevalence estimates of asthma in neighbourhoods use collateral morbidity and covariates observed in neighbourhoods, as well as observed asthma prevalence for GP practice areas. In this application the data consist of

(a) counts of patients (in the financial year 2010–2011) with diagnosed asthma for GP practice areas (source areas); these counts are released as a single total for each GP practice area with no breakdown by sex or age;
(b) collateral information on asthma hospital admissions for neighbourhoods (also counts) in 2010–2011; morbidity indicators such as this are taken as reflexive of prevalence for neighbourhoods (target areas), and
(c) an air quality index for neighbourhoods in 2008 (target areas), based on levels of nitrogen dioxide, particulates, sulphur dioxide and benzene. The index was developed by the Geography Department at Staffordshire University using data from the UK National Air Quality Archive [7].

There is a slight time period discrepancy between the air quality data and the health datasets, but air quality differences between areas are likely to be broadly stable over such a period—as evidenced by stability in the London Atmospheric Emissions Inventory (LAEI) concentrations [8]. Also available are 2010 population estimates for both GP areas and neighbourhoods, so that expected prevalence totals (populations multiplied by the region-wide prevalence rate) can be obtained for both source and target areas. In other applications there might be more than one type of collateral indicator reflecting prevalence (e.g., mortality counts as well as hospitalizations) and more than one ecological predictor.

## 3. Methods

### 3.1. Model for Prevalence Data

This section considers modelling observed and latent prevalence data, for GP areas and neighbourhoods respectively. We consider first the model assumed for prevalence in GP areas. Consider total observed cases *y_i_* of disease in GP areas with geographic centroids (*s_1_*,.., *s_N_*) (sometimes called eastings and northings), where *s_i_* = (*s_1i_*, *s_2i_*). These are assumed to be Poisson distributed:

*y_i_* ~ *Po*(*E_i_ρ_i_*) (1)

where the *E_i_* represent expected prevalence totals, and the *ρ_i_* then have a relative risk interpretation. Specifically the *ρ_i_* are interpretable as relative prevalence rates (with average 1). 

Variation in relative prevalence is assumed to be partitioned between that due to covariates *X_i_*, that due to a spatial process *z_1_*(*s_i_*) (the specification of which is elaborated below), and a residual random term *e_i_* to account for Poisson over-dispersion. Over-dispersion is frequently encountered in practice for health count data [9]. Because relative risks are necessarily positive, a log-link is used so that:

log(*ρ_i_*) = *X_i_β* + *z_1_*(*s_i_*) + *e_i_,* (2)

where *β* are regression parameters, and the residuals are assumed to be normally distributed with an area-specific variance *V_i_*:

*e_i_* ~ *N*(0,*V_i_*) (3)

Variances may be related to population size in source areas (e.g., greater residual variation for smaller populations) and are modelled as:

log(*V_i_*) = *δ*_1_ + *δ*_2_*E_i_* (4)

Note that, as in the present study, ecological predictors of prevalence (e.g., air quality) may not necessarily be observed for source areas (here GP areas). A subscript *L* (denoting latent data) is added to indicate this, so that Model 1 becomes:

log(*ρ_i_*) = *X_Li_β* + *z_1_*(*s_i_*) + *e_i_* (5)

The spatial process *z_1_*(*s_i_*) is distinct from another process used to interpolate predictor values *X_Li_* to GP areas (see Section 3.2), and is modelled by a convolution of a random effect and a smoothing kernel. The random effects, denoted *w*_1*j*_, are sampled at a discrete grid of *J < N* points at geolocations *u_j_* = (*u*_1*j*_, *u*_2*j*_). The interpolated spatial effect at points *s_i_* is then obtained as:

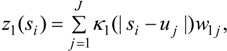
(6)
where *κ*_1_(|*s* − *u*|) is a two-dimensional smoothing kernel, and *d* = |*s* − *u*| represents distance.

An analogous model is assumed for modelling neighbourhood prevalence counts. Although such counts are latent rather than observed, expected neighbourhood prevalence totals *E_k_* can be calculated. Then for *k* = *1*,.., *K* neighbourhoods with centroids *t_k_* = (*t*_1*k*_,*t*_2*k*_), and expected prevalence counts *E_k_*, latent neighbourhood prevalence counts are distributed as:

*y_Lk_* ~ *Po*(*E_k_ρ_k_*), (7)

with risk model involving observed ecological covariates *X_k_*, namely:

log(*ρ_k_*) = *X_k_β* + *z*_1_(*t*_k_) + *e_k_,* (8)

and with the spatial process *z*_1_ defined with respect to *t_k_* as:

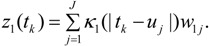
(9)


The same variance model used for source area random effects *e_i_* is assumed for neighbourhood random effects *e_k_*, so that:

*e_k_* ~ *N*(0,*V_k_*), (10)

log(*V_k_*) = *δ*_1_ + *δ*_2_*E_k_* (11)

### 3.2. Using Collateral Morbidity and Ecological Covariate Data

There are two sources of collateral information: indicators that reflect prevalence, and indicators that may cause variations in prevalence (*i.e.*, reflexive indicators and formative indicators respectively). Collateral reflexive indicators of morbidity are provided in the case study by neighbourhood asthma hospitalisations. These are taken as reflexive of prevalence in that higher prevalence is likely to be associated with higher asthma hospitalisation rates: if a neighbourhood population of a given size has a higher number of asthmatic patients, then other things equal it will tend to have higher number of inpatient admissions with asthma as the precipitating condition. Of course, in practice, there may be additional factors affecting hospitalisation levels, though these are likely to some extent to influence hospitalisation indirectly by affecting prevalence levels. These factors might include smoking levels, poverty rates, or urban-rural setting.

Consider hospitalisation data in the form of counts *M_k_* for neighbourhoods *k*. Then observed hospitalisation counts are taken as Poisson:

*M_k_* ~ *Po*(*G_k_ν_k_*), (12)

where *G_k_* are expected hospitalisations, and relative hospitalisation risks *ν_k_* are modelled as a function of relative neighbourhood prevalence:

log(*ν_k_*) = *γ*_1_ + *γ*_2_*ρ_k_* (13)

It is expected that *γ*_2_ > 0 on the basis that higher neighbourhood asthma prevalence rates *ρ_k_* will be associated with higher risks *ν_k_* of hospitalisation with asthma diagnosis. This is a substantive expectation, not a constraint explicitly applied in the model.

Reflexive indicators provide additional information relevant to interpolating neighbourhood prevalence. So also do formative indicators, namely area risk factors for prevalence. A number of studies indicate that asthma prevalence may be increased by air pollution [10,11]. The air quality index in the present analysis is a continuous index, and is available (*i.e.*, observed) for the target areas (neighbourhoods) *k* = *1*,.., *K*, but not available for GP areas. 

Thus a second kernel process *z*_2_(*s*), defined using the same grid points *u_j_* as *z*_1_(*s*), is used to spatially interpolate air quality values to GP areas *i* = *1*,.., *N*. Thus:

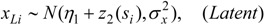
(14)

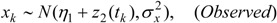
(15)

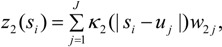
(16)

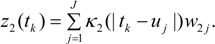
(17)


This spatial process *z*_2_() can be denoted the risk factor process, as opposed to the prevalence spatial process *z*_1_().

### 3.3. Assumptions Regarding the Spatial Process

The fit of the model to the observed data (asthma prevalence counts for GP areas, together with asthma hospitalisations, and air quality data for neighbourhoods) may be affected by assumptions regarding the kernels *κ*(*d*) and the random processes *w_j_*. Inferences about the spatial pattern of the interpolated neighbourhood asthma prevalence rates may also be sensitive to model specification (see Section 3.4).

While a bivariate normal kernel (with scale parameter *α*), namely:

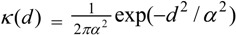
(18)
is often assumed, other bivariate kernel functions have been discussed, for example in the literature on seed dispersal [12,13]. For example, a two dimensional exponential has the form:

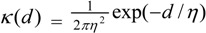
(19)
and a two dimensional Student *t* has the form:

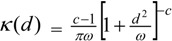
(20)


For the random process *w_j_*, possible alternatives to a normal process include Student *t*:

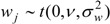
(21)
(with degrees of freedom *ν*), or *w_j_* taken as spatially correlated over points in the discrete grid [14].

Regarding estimation, it may be noted that if the parameters of the random process *w* and of the kernel function *κ*(*d*) are both taken as unknown, they will be confounded. For unique identification, one can adopt standardised forms in either the kernel or the random process, with the unknown parameter(s) then remaining in the other component. 

### 3.4. Assessing Alternative Spatial Process Assumptions

Overall fit to the observed data (prevalence *y*, admissions *M*, pollution *x*) is here based on the Deviance Information Criterion [15]. Sensitivity of inferences to model assumptions is also important. In particular, for assessing spatial pattern in the interpolated neighbourhood prevalence rates, one may compare models in terms of:

(a) localised hot spot probabilities of high asthma risk (excess risk in each neighbourhood without regard to risk in surrounding neighbourhoods),
(b) clustering of excess asthma risk, namely elevated risk in both neighbourhood *k* and the surrounding neighbourhoods.

To this end one may define binary indicators:

*J_k_* = *I*(*ρ_k_* > 1) (22)

where *I*(*R*) = 1 if condition *R* pertains and *I*(*R*) = 0 otherwise. The Bayesian estimation process can be used to provide an estimate of probabilities *Pr*(*ρ_k_* > 1|*y*,*M*,*x*) that relative prevalence risk is above average. The classification of areas as having excess localised risk may be based on a threshold such as 0.9 or 0.8 [16], namely:

*Pr*(*ρ_k_* > 1|*y*,*M*,*x*) > 0.8 (23)

To assess spatial clustering in excess asthma risk, one may monitor co-occurrence of excess risk in both neighbourhood *k*, and in the *L_k_* neighbourhoods in its surrounding locality *A_k_*. Thus consider the indicators:

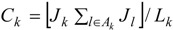
(24)


Then *C_k_* is a probability indicator of a high risk cluster centred on area *k*, since higher values for *C_k_* occur as more adjacent areas have high risk *J_l_* = 1 (for *l* ∈ *A_k_*) in combination with high risk in area *k* itself (*J_k_* = 1).

To compare how different model specifications (e.g., kernel and grid random process choices) affect the resulting interpolated spatial prevalence pattern, one may cross-tabulate totals of neighbourhoods classified (or not) as localized high risk or as high risk cluster centers.

## 4. Analysis and Results

### 4.1. Analysis Framework

**Figure 1:**
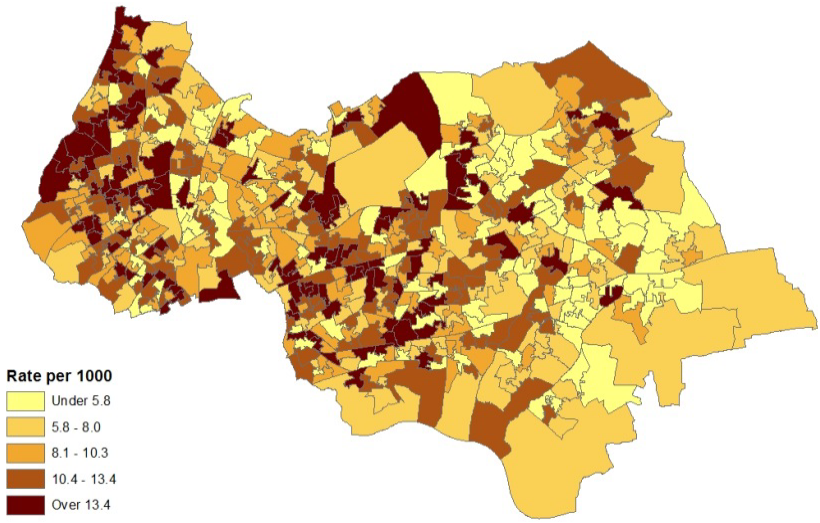
Asthma hospitalisations, rates per 1,000, LSOAs, outer NE London.

**Figure 2:**
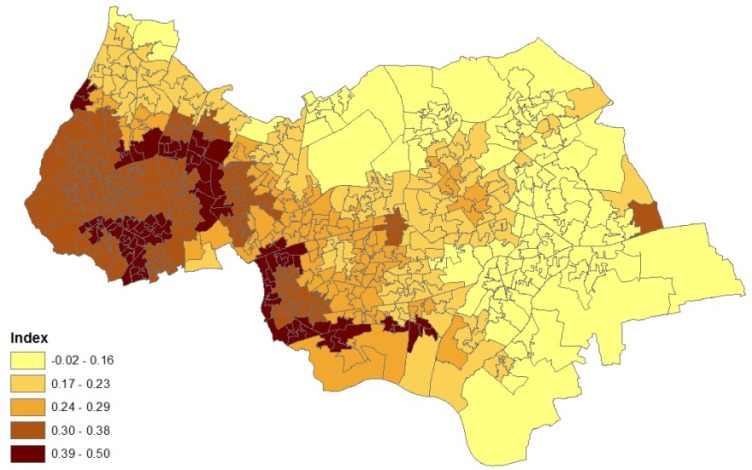
Index of low air quality, LSOAs, outer NE London.

The model set out above is applied to spatial interpolation of asthma and pollution, and to estimation of the impact of pollution on prevalence in Outer NE London. Bayesian estimation is carried out using the WINBUGS package. Observed asthma data (ICD10 codes J45-J46) consist of prevalence counts *y_i_* for GP practice areas in 2010–2011. These data provide source area observations to make interpolated prevalence estimates for neighbourhoods (target areas). Additional collateral data are asthma hospitalisations *M_k_* for neighbourhoods, and air quality indices *x_k_* (log transformed), also for neighbourhoods. Note that higher *x* values denote worse air quality. Figure 1 and Figure 2 contain maps of hospitalization rates (crude rates per 1,000 population) and the logged air quality indices. 

There are *N* = 189 GP areas, with an average population of 5,380, and *K* = 562 neighbourhoods (LSOAs). Neighbourhood and GP area locations are represented by population weighted centroids. There are *J* = 84 points at 2 km spacing in the discrete grid covering the region. 

Of importance for practical application of the methodology of Section 3 is stability or otherwise of inferences regarding the distribution of, and spatial patterning in, the interpolated asthma prevalence rates in neighbourhoods. Four alternative models are applied differing in kernel form (bivariate normal *vs*. bivariate exponential) and in the random process (normal *vs*. Student *t*). The same assumption regarding the kernel-process combination is applied both to the prevalence process *z*_1_ and the risk factor process *z*_2_. There is a wide range of possible model options: different kernels, different form of random grid effect (*w_j_*), and differing regression forms for *ρ* on *x*. The selected four models are intended as a representative subset of the possibilities. 

All four models assume standard kernels and a linear regression effect of ecological covariates *x* on prevalence risk *ρ*. Thus model 1 combines a standard normal kernel (*α* = 1) with a normal process *w_j_* ~ *N*(0,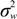) with unknown variance. Model 2 combines a standard exponential kernel (*η* = 1) with a normal process *w_j_* ~ *N*(0,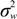). As mentioned by Clark *et al*. [13], exponential kernels allow for more leptokurtic (more peaked and fat tailed) densities than normal kernels, and so exponential kernels may represent a more flexible assumption for latent prevalence rates *ρ* and air quality rates *x*. Model 3 combines a standard normal kernel with a Student *t* process with five degrees of freedom *w_j_* ~ *t*(0, 5, 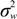), and Model 4 combines a standard exponential kernel with a Student *t* process with five degrees of freedom. The Student *t* with low degrees of freedom allows for heavier tails than the normal. 

Bayesian inference requires that prior densities on parameters be specified. Since there is no extensive prior evidence, relatively diffuse options are chosen. Gamma priors with scale 1 and index 0.001 are assumed for unknown precisions, and normal priors with mean 0 and variance 1,000 are assumed for regression parameters {*β*,*γ*,*δ*}. Inferences are based on the second halves of two chain runs of 25,000 iterations, with convergence assessed using Brooks-Gelman criteria [17]. 

While stability of inferences is an important aspect in assessing the interpolated prevalence data, fit to the three observed datasets is, of course, also central. Table 1 uses the DIC criterion to assess how well the method estimates the observed small-area counts or rates, whether obtained on one area frame (GP service areas) or the other (residential neighbourhoods). The DIC values are based on the Poisson deviance for *y_i_* (prevalence counts observed over GP service areas) and *M_k_* (hospitalisations observed over neighbourhoods) namely:

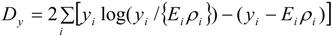
(25)
and:

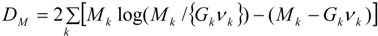
(26)


The deviance for the air quality data *x_k_* (observed over neighbourhoods) is:

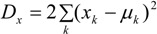
(27)
where *µ_k_* = *η*_1_ + *z*_2_(*t_k_*). 

### 4.2. Results

Defining a composite measure of fit is complicated by the different scales of the three sets of observations (and hence deviances), but it can be seen from Table 1 that no model provides a best fit across all observations. The best fit for the *y*-data (generated by modelled prevalence rates *ρ_i_* in GP areas) and *M*-data is provided by a normal kernel *κ* combined with a normal process *w*, while the best fit for the *x*-data is provided by an exponential kernel combined with Student *t* errors. Such results illustrate that default choices such as normal kernels may not necessarily provide best performance. 

**Table 1:** Deviance information criteria.

	Observed Data		
Model	*y*	*M*	*x*
1	376.5	1,096.9	3.234
2	378.1	1,107.7	3.223
3	377.5	1,104.1	3.220
4	379.2	1,098.4	3.207

Observed data: *y*, asthma prevalence counts for 189 GP practice areas; *M*, asthma hospitalisations for 562 neighbourhoods.

Table 2 summarises estimates of the main structural parameters across sub-models. These are *β*_1_ and *β*_2_ in the GP area and neighbourhood prevalence equations:

log(*ρ_i_*) = *β*_1_ + *β*_2_*x_Li_* + *z*_1_(*s_i_*) + *e_i_* (28)

log(*ρ_k_*) = *β*_1_ + *β*_2_*x_k_* + *z*_1_(*t_k_*) + *e_k_* (29)

the “reflexive effect” parameter *γ*_2_ in the model for neighbourhood hospitalisations:

log(ν_k_) = γ_1_ + γ_2_ρ_k_ (30)

and the heteroscedasticity parameters in the log-variance equations:

log(*V_i_*) = *δ*_1_ + *δ*_2_*E_i_* (31)

log(*V_k_*) = *δ*_1_ + *δ*_2_*E_k_* (32)

**Table 2:** Posterior summary (means and quantiles), structural parameters.

Parameter	Interpretation	Model	Mean	2.5%	5%	95%	97.5%
*β*_1_	Prevalence Model Intercept	1	−0.25	−0.35	−0.33	−0.17	−0.16
		2	−0.22	−0.32	−0.31	−0.11	−0.08
		3	−0.23	−0.30	−0.29	−0.15	−0.14
		4	−0.23	−0.33	−0.32	−0.14	−0.12
*β*_2_	Prevalence Model, Pollution Effect	1	0.31	−0.02	0.03	0.58	0.61
		2	0.22	−0.26	−0.14	0.49	0.52
		3	0.25	−0.09	−0.03	0.48	0.51
		4	0.25	−0.08	−0.03	0.58	0.65
*γ*_1_	Hospitalisation Model Intercept	1	−1.82	−2.06	−2.04	−1.62	−1.60
		2	−1.78	−2.05	−2.01	−1.56	−1.53
		3	−1.87	−2.06	−2.03	−1.70	−1.68
		4	−1.88	−2.06	−2.03	−1.75	−1.73
*γ*_2_	Hospitalisation Model, Prevalence Effect	1	0.38	0.33	0.33	0.43	0.43
		2	0.36	0.30	0.31	0.42	0.43
		3	0.38	0.34	0.35	0.42	0.42
		4	0.38	0.35	0.36	0.42	0.43
*δ*_1_	Heteroscedasticity Model, Intercept	1	−3.37	−5.32	−5.04	−1.73	−1.48
		2	−2.63	−4.37	−4.19	−2.67	−0.99
		3	−2.55	−4.42	−4.16	−1.32	−1.01
		4	−2.27	−4.37	−4.12	−0.58	−0.29
*δ*_2_	Heteroscedasticity Model, Slope	1	0.03	−0.34	−0.29	0.35	0.39
		2	−0.09	−0.43	−0.40	−0.09	0.21
		3	−0.12	−0.41	−0.36	0.17	0.24
		4	−0.16	−0.52	−0.48	0.19	0.23

It can be seen that all models agree on the positivity of *γ*_2_, namely that asthma hospitalisation rates in neighbourhoods increase in line with neighbourhood prevalence of asthma. All models also provide closely similar results on the level of prevalence (the parameter *β*_1_).

However, results are more equivocal concerning the effect of pollution (poor air quality) on asthma prevalence. Posterior means for this coefficient are all positive, ranging from 0.22 to 0.31, but a significant effect, namely an entirely positive 90% credible interval, is only apparent under Model 1. There is also no clear evidence that variances of unstructured residuals *e_i_* and *e_k_* are related to population size.

To assess stability in inferences about the spatial pattern of asthma risk, the four models are compared in terms of co-location of neighbourhoods identified as having high localized risk, namely:

*Pr*(*ρ_k_* > 1|*y*,*M*,*x*) > 0.8 (33)

and in terms of co-location of cluster centres, defined by the probabilities *C_k_*. The probabilities that neighbourhoods are classed as cluster centres has a lower threshold of 0.25, as this classification requires coincidence of one or more *J_l_* = *I*(*ρ_l_* > 1) = 1 (for *l* ∈ *A_k_*) together with *J_k_* = 1.

It can be seen from Table 3 that a normal kernel combined with a normal discrete process (Model 1) leads to smaller totals of neighbourhoods classed as local high asthma risk or as cluster centres than the other models. However, cross-tabulation of co-located high risk areas or cluster centres (in the lower two subtables of Table 3) show that all areas classed as locally high risk or as cluster centres under Model 1 are also classed as such by the other models. 

**Table 3:** Classification of asthma risk in neighborhoods.

Number of Neighbourhoods (from *K* = 562)			
Model		Total Neighbourhoods with Local Exceedance Probabilities > 0.8	Total Neighbourhoods with Cluster Centre Probabilities > 0.25
1		66	68
2		75	90
3		74	87
4		80	89
**Colocation of local exceedance and cluster centre classifications **			
*Local exceedance*		*Model* 1	
Model		No	Yes
2	No	487	0
	Yes	9	66
3	No	488	0
	Yes	8	66
4	No	482	0
	Yes	14	66
*Cluster centres*		*Model* 1	
Model		No	Yes
2	No	472	0
	Yes	22	68
3	No	475	0
	Yes	19	68
4	No	473	0
	Yes	21	68

Table 4 considers distributional features of neighbourhood asthma prevalence as indicated by posterior mean asthma prevalence rates from the four models (expressed as percentage rates). There is close agreement between Models 2–4, but slightly lower mean prevalence, and lower 95th and 99th percentiles under Model 1. 

**Figure 3:**
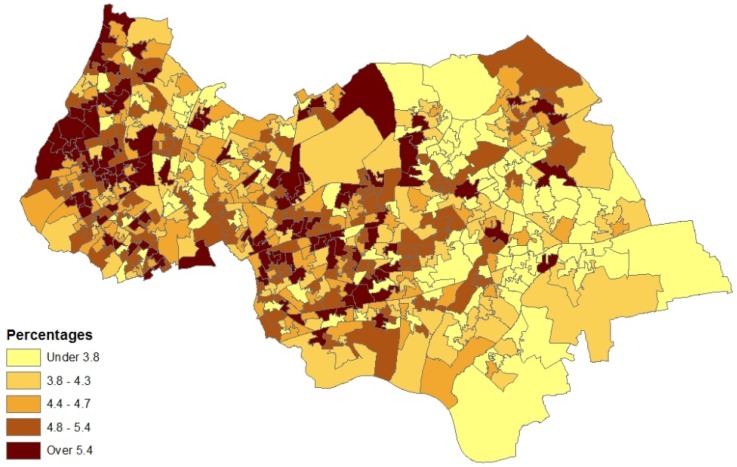
Asthma prevalence (%), LSOAs, outer NE London.

Figure 3 depicts the distribution of asthma prevalence over the Outer NE London region, using Model 1 estimates. There is a close correspondence between prevalence and hospitalization rates (Figure 1), even though the latter are simple crude rates, so providing consistent evidence on the asthma burden. 

**Table 4:** Neighbourhood asthma prevalence (percentage rates).

Distributional Characteristics				
	1	2	3	4
Mean	4.58	4.65	4.65	4.65
Median	4.50	4.56	4.57	4.58
Skewness	0.44	0.48	0.44	0.43
1st percentile	2.73	2.76	2.70	2.74
5th percentile	3.14	3.12	3.14	3.12
95th percentile	6.36	6.53	6.50	6.47
99th percentile	6.99	7.24	7.19	7.13

## 5. Concluding Remarks

Profiles of neighbourhood prevalence of major chronic diseases such as asthma are important for public health priority setting. In the UK, the prevalence of a number of chronic diseases is recorded by General Practitioner teams for the areas they serve, but this data is not provided for neighbourhoods. 

To make spatially interpolated estimates of prevalence for neighbourhoods one might simply rely on using geographic location data and apply a multivariate Gaussian process. However, such a procedure neglects subsidiary information about neighbourhood disease prevalence. First, there may be reflexive indicators (e.g., neighbourhood level hospitalisations or deaths due to the disease being considered). Second there may be observations for neighbourhoods on ecological risk factors for prevalence of the disease.

This paper has demonstrated how such information can be utilised within an encompassing model for prevalence, reflexive morbidity and ecological risk indices, with a risk interpolation model being necessary if the risk variable (here air quality) is only observed for neighbourhoods. Interpolation is based on a kernel convolution methodology combining different possible assumptions about kernel form and random process. 

Among the four models considered, none has a clear best fit to the observed data. There is some sensitivity in inferences about interpolated prevalence in neighbourhoods (the target areas), the main differences being between Model 1 (combining a normal kernel and a normal random process) compared to the other models. However, inferences are broadly stable about the distributional form and spatial patterning between the four models. Such stability supports use of the methodology for other regions and diseases.

Other techniques are available for spatial interpolation, but are not adapted to including the interconnected ancillary variables used here in an overarching interpolation methodology which recognizes their interdependence. GIS packages could be used to interpolate asthma neighbourhood prevalence rates using GP area prevalence data, and to separately interpolate pollution rates to GP service areas using neighbourhood pollution data. For example, the ArcGIS package uses deterministic interpolation techniques such as inverse distance weighted interpolation, as well as geostatistical methods [18]. This type of interpolation would not take into account additional information provided by ancillary variables such as neighbourhood asthma hospitalisations. 

Off-the-shelf interpolation techniques will also not typically provide the full range of inferences possible with the Bayesian simulation methods used in the current paper. Thus as well as estimating asthma prevalence, one may use the simulation (see Section 3.4) to estimate the probabilities of elevated risk in a neighbourhood, and also whether or not elevated risk characterizes both a particular area and its neighbours—that is to distinguish between spatial clustering and spatial outliers [19]. In broader terms, Bayesian estimation has benefits in fitting relatively complex random effects models such as the one in this paper, whereas classical estimation would have to involve approximate numerical integration methods (e.g., quadrature), and may not necessarily even be feasible. 

It is important for public health priority setting to identify areas with excess risk and also spatial clustering of excess risk, as evidence of either pattern may provide support for targeted interventions, resourcing or health promotion campaigns. One might apply the methodology here to other respiratory diseases where interpolation is relevant, and where ancillary variables are also generally available. Examples are other types of respiratory disease prevalence data available only for GP service areas (such as COPD prevalence), or when incidence data (e.g., for lung cancer) is only released for relatively aggregated areas. For example, in the UK, lung cancer incidence data are released for middle level super output areas (of which there are currently 6,791 across England), rather than LSOAs (of which there are 32,844). The methods in this paper can be adapted to this type of application, namely to interpolate lung cancer incidence to LSOAs. Having identified neighbourhoods with jointly elevated risk across conditions (just as Table 3 and Figure 3 identify excess risk asthma risk), one may then ascertain whether common precipitating influences are present.
